# Democratized image analytics by visual programming through integration of deep models and small-scale machine learning

**DOI:** 10.1038/s41467-019-12397-x

**Published:** 2019-10-07

**Authors:** Primož Godec, Matjaž Pančur, Nejc Ilenič, Andrej Čopar, Martin Stražar, Aleš Erjavec, Ajda Pretnar, Janez Demšar, Anže Starič, Marko Toplak, Lan Žagar, Jan Hartman, Hamilton Wang, Riccardo Bellazzi, Uroš Petrovič, Silvia Garagna, Maurizio Zuccotti, Dongsu Park, Gad Shaulsky, Blaž Zupan

**Affiliations:** 1 Faculty of Computer and Information Science, University of Ljubljana, 1000 Ljubljana, Slovenia; 2 Department of Molecular and Human Genetics, Baylor College of Medicine, Houston, TX 77030 USA; 3 Faculty of Engineering, University of Pavia, 27100 Pavia, Italy; 4 Biotechnical Faculty, University of Ljubljana, 1000 Ljubljana, Slovenia; 5 Department of Molecular and Biomedical Sciences, Jožef Stefan Institute, 1000 Ljubljana, Slovenia; 6 Department of Biology and Biotechnology, University of Pavia, 27100 Pavia, Italy

**Keywords:** Data mining, Image processing, Machine learning

## Abstract

Analysis of biomedical images requires computational expertize that are uncommon among biomedical scientists. Deep learning approaches for image analysis provide an opportunity to develop user-friendly tools for exploratory data analysis. Here, we use the visual programming toolbox Orange (http://orange.biolab.si) to simplify image analysis by integrating deep-learning embedding, machine learning procedures, and data visualization. Orange supports the construction of data analysis workflows by assembling components for data preprocessing, visualization, and modeling. We equipped Orange with components that use pre-trained deep convolutional networks to profile images with vectors of features. These vectors are used in image clustering and classification in a framework that enables mining of image sets for both novel and experienced users. We demonstrate the utility of the tool in image analysis of progenitor cells in mouse bone healing, identification of developmental competence in mouse oocytes, subcellular protein localization in yeast, and developmental morphology of social amoebae.

## Abstract (web summary)

Deep learning approaches for image preprocessing and analysis offer important advantages, but these are rarely incorporated into user-friendly software. Here the authors present an easy-to-use visual programming toolbox integrating deep-learning and interactive data visualization for image analysis.

## Introduction

Deep learning^1^ has revolutionized the field of biomedical image analysis. Conventional approaches have used problem-specific algorithms to describe images with manually crafted features, such as cell morphology, count, intensity, and texture. Feature learning with deep convolutional neural networks is implicit, and training the network usually focuses on particular tasks, such as breast cancer detection in mammography^2^, subcellular protein localization^3^, or plant disease detection^4^. Training a deep network usually requires a large number of images, which limits its utility. For example, the classifier for plant disease detection by Mohanty et al.^4^ was trained on 54,306 images of diseased and healthy plants, and the yeast protein localization model by Kraus et al.^3^ was inferred from 22,000 annotated images, but not everyone who could benefit from image analysis has so many well-annotated images.

Machine learning on images does not always need training on a closely related set of images. Just like our visual cortex can adapt to the analysis of many scenes and images, a deep network pre-trained on a sufficiently large number of diverse images may infer useful features from a broad range of new image sets. This idea is based on transfer learning^5^, a machine-learning technique that stores the knowledge obtained from one problem in a trained model and applies it to another problem, which may be quite different. A typical deep network for image analysis^6,7^ contains convolutional layers that identify structural features of the images followed by fully connected layers that combine the features and find interactions between them. When applied to classification, the network nodes of the penultimate layer contain information about the most informative combination of image features, and the final layer includes one node for each image class that reports on class probability. For transfer learning, we can repurpose a deep network trained on one set of images through retraining on another collection of images. In the purest form of knowledge transfer, we need to retrain only the last layer of the network: images from the new collection are represented with feature vectors inferred by the existing deep model, and their relations with the image class are inferred by applying a machine-learning method such as logistic regression.

A successful example of such deep network repurposing was, for instance, proposed for diagnosis of dermatology^8^, where the authors started with an existing deep network and re-trained it to classify skin cancer. As a starting model that embeds images into feature space, the authors used Google’s InceptionV3^6^, a convolutional 48-layered neural network that was trained on 1.2 million images from the ImageNet repository. ImageNet includes images depicting real life objects, such as vehicles (locomotive, amphibian, minivan), tools (shovel, screwdriver), and animals (tick, tarantula, bee), most of which are not similar to images from the field of dermatology, or even molecular biology research images that we later use in cases that demonstrate our proposed tool. To classify skin cancer, a part of InceptionV3 was re-trained over 120,000 clinical images that included 3374 dermoscopy images. The resulting system achieved dermatologists-level classification accuracy. A similar approach was proposed to classify in situ hybridization images of the fruit fly^9^, where an existing deep model trained on ImageNet images was repurposed for classification of over 23,000 fruit fly images at different stages of embryogenesis. Transfer learning has also been successfully used in other fields, such as the analysis of images from an electron microscope^10^, X-ray computed tomography^11^, and pathology^12^. Just like we, humans, can adapt our visual recognition to any new image classification task through additional training, the above examples show that artificial intelligence systems can adapt trained models to new tasks. Transfer learning was first proposed in the late 1990s^13,14^, but it is being increasingly adopted in image analytics with the utility of deep models. The potential of transfer learning is being well recognized in the biomedical sciences^15^.

Here, we propose a visual programming approach to image analytics, where the users can combine image embedding by pre-trained deep models with clustering and classification. Our tool supports the execution of essential data mining functions on images in an easy-to-use framework, where common data analysis tasks can be conceived and executed within minutes. The proposed tool also makes image analytics accessible to anyone that can spare an hour for training, or even only minutes for watching the educational videos that we provide together with the tool. While the proposed framework is general and can consider any type or class of images, we focus here on biomedicine and demonstrate the utility of the tool for analysis of images from molecular and cell biology.

## Results

### Image analytics by visual programming

We have designed a toolbox for image analytics that features visual programming. The toolbox supports users in assembly of analysis workflows that are comprised of components that load the images, embed them into a vector space, and analyze these image profiles to infer image clusters or classifications. The toolbox is based on Orange data mining^16,17^, a general-purpose data analysis framework that already includes components for clustering, classification, and interactive data and model visualizations. Image-specific extension described in this paper is packaged in Orange’s add-on for image analytics. Both Orange and the proposed extension are provided as open-source and are freely available through Orange’s home page at http://orange.biolab.si.

**Fig. 1:**
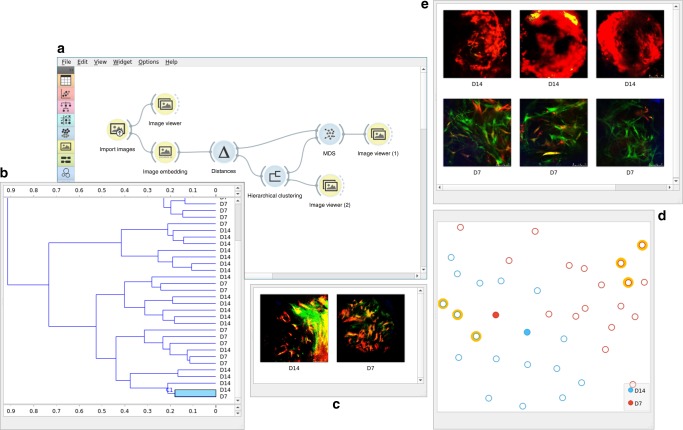
Unsupervised analysis of bone healing images. **a** The data analysis workflow starts with importing 37 images from a local folder. The images can be viewed in the Image Viewer widget (not shown) and are passed to the Image Embedder, which was set to use Google’s InceptionV3 deep network. We computed the distances between the embedded images and presented them as a dendrogram (**b**) with the Hierarchical Clustering widget. The clusters corresponded well to the time (days) post injury (D7 and D14), with a few exceptions. One such exception was a branch of two images highlighted in the dendrogram (**b**) and shown in the Image Viewer (2) (**c**). Image distances were also given to the multi-dimensional scaling widget (MDS), that also exhibits separation between bone healing samples at different times as depicted in different colors. Three representative MDS points from D7 and D14 were selected manually (data points with orange boundaries) (**d**) and the images are shown in the Image Viewer (1) (**e**). The two images highlighted in the dendrogram (**b**) were also passed to the MDS widget as a data subset. They are visualized as filled dots in this data projection (**d**) and they appear close to each other because of their similarity. This figure illustrates how a biologist may explore the data after clustering—first focusing on the misclassified samples and looking at the images and then selecting some of the best classified images as a point of reference for further exploration

Data analysis in Orange is implemented through workflows. A workflow (see Fig. 1 for example) consists of widgets—components that can process, model, or visualize the data. Widgets accept data as their input and display or send results as their output. Data analysis workflows in Orange are defined by the selection of widgets and connections between them. For instance, the workflow in Fig. 1 loads a set of images from the chosen directory, represents images with feature vectors through embedding, estimates the distances between these vectors and hence between the images, and uses the computed distances in clustering and visual depictions of image similarities in the multi-dimensional scaling plot. Users can monitor the execution of every step of the Orange workflow and inspect every intermediate result. For instance, they can check the images that have been loaded (Image Viewer connected to Load Images in the workflow from Fig. 1), visualize the result of hierarchical clustering in a dendrogram, and even visualize the selection of images from a specific branch of the dendrogram or from a section of points in the multi-dimensional scaling plot. The users can also inspect the raw data coming from embedders, or feature profiles of the selected images in the dendrogram (achievable by connecting the Data Table widget to any of the widgets in the workflow; for brevity not shown in Fig. 1). The ability to check and inspect the results at every step of the analysis pipeline helps the users in gaining confidence in results and familiarity with the analysis procedures. It also provides a tool for educators to explain different analysis steps to potential trainees.

### Interactive data visualizations

Widgets in Orange are interactive, and they can immediately transmit the results upon any change in the widget parameters or any change in the selection of elements in their graphical presentations. For instance, the hierarchical clustering widget from Fig. 1 allows users to select the branch of the constructed dendrogram. Upon selection, the Hierarchical Clustering widget outputs the data corresponding to the selected branch, which is fed into the image visualization widget (Image Viewer (2) in Fig. 1). Any change in the selection of branches in the dendrogram propagates through the workflow and updates the content of any of the downstream widgets, instructing, for instance, Image View (2) to immediately display the images that are pertinent to the user’s selection in the dendrogram.

### Case studies

**Fig. 2:**
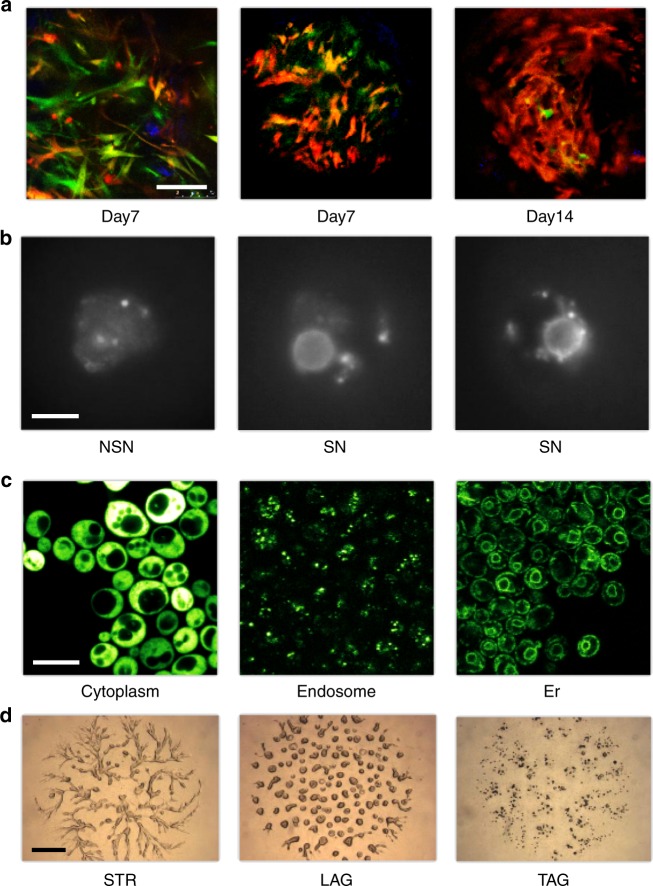
Example images considered in our pilot study encompass diverse fields in biomedicine. **a** Bone-fracture repair involves skeletal stem cells. The images in this example are from mice that were the progeny of a cross between mice carrying Mx1/Tomato (red), which is a skeletal stem/progenitor cells marker, and mice carrying αSMA-GFP or Nestin-GFP (green), which are mesenchymal cell markers. The bones were injured and images were taken in vivo at 7 days and 14 days after injury, when critical events in the early repair process occur. **b** Chromatin organization (Hoechst staining) in the nucleus of mouse fully grown antral oocytes. Depending on their chromatin organization, oocytes are classified as surrounded nucleolus (SN), with a ring of heterochromatin surrounding the nucleolus and not surrounded nucleolus (NSN) oocytes, with a more dispersed chromatin not surrounding the nucleolus. SN oocytes are developmentally competent, whereas NSN oocytes are incompetent^18^. **c** Protein localization in budding yeast—fluorescence micrographs of GFP-fusion proteins localized to the cytoplasm, endosome or endoplasmic reticulum (er) as indicated. **d** Images of *Dictyostelium discoideum* cells at different developmental stages—streaming (STR), loose aggregate (LAG), and tight aggregates (TAG). Scale bars are 100 μm (**a**), 10 µm (**b**), 5 µm (**c**), or 1 mm (**d**). See Supplementary Note 1 for detailed description of the image sets

We illustrate the visual programming approach and the interactive visualizations through the analysis of highly diverse multi-color image sets that include stem/progenitor cells in bone healing at various mouse ages (3–12 months), identification of developmentally competent or incompetent mouse oocytes, subcellular protein localization in yeast, and developmental morphology of the social amoeba *Dictyostelium discoideum* (Fig. 2). The phenotypes reflected in the images were assessed by domain experts. For instance, the oocytes were classified as developmentally competent whenever a ring of Hoechst-positive chromatin was observed surrounding the nucleolus or as developmentally incompetent when this ring was not evident and the chromatin appeared more diffuse^18^. The phenotypes of the developing social amoebae were determined by visual inspection based on morphological features, such as the presence of cell aggregates or streams, according to terms from the *Dictyostelium* phenotype ontology in dictyBase (http://dictybase.org). Subcellular localization of proteins in budding yeast was reported by curators of the YPL + database (http://yplp.uni-graz.at). We considered the possibility that manual curation and classification in biomedicine might contain errors, so we used data mining methods that can handle noise and inconsistencies.

Typical tasks in biomedical data mining include clustering, data projection (unsupervised data mining), and development of prediction models (supervised data mining). Orange provides a simplified interface to these tasks that can be executed with workflows that consist of only a few data processing and visualization elements^16^. Figure 1 shows a workflow for unsupervised analysis of in vivo imaging of skeletal stem cells during mouse bone healing. The workflow loads the images, embeds them in vector space, computes distances between the image vectors and performs unsupervised mining through clustering and data projection. Regardless of the in vivo morphological diversity of stem cells due to sex, age, and location, we consistently found good separation of image classes during their biological responses. This finding illustrates that the general morphology and cellular responses of stem/progenitor cells remain biologically interpretable and that the method can be used for analyzing images from vastly diverse domains of biology with high fidelity. The workflow in Fig. 1 was also used to analyze the other three sets of images (Supplementary Note 1).

**Fig. 3:**
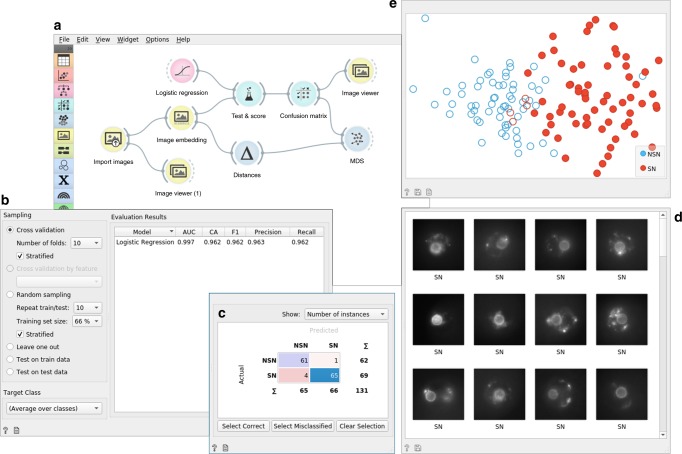
Supervised data analysis of 131 mouse oocyte images with surrounded (SN) or not surrounded (NSN) chromatin organization. **a** The data analysis workflow first imports the data from the local directory where images are stored in respective subdirectories named SN and NSN. Vector-based embedding passes the data matrix to a cross-validation widget (Test and Score) that accepts a machine-learning method (logistic regression) as an additional input. The Test and Score widget displays the cross-validated accuracy (area under ROC curve—AUC, classification accuracy—CA, and harmonic average of the precision and recall—F1 score) (**b**) and sends the evaluation results to the Confusion Matrix widget (**c**). The Confusion Matrix widget provides information on misclassification. In this example, 65 of the 69 SN oocytes were classified correctly. Selection of this particular cell in the Confusion Matrix triggers sending these images and their descriptors further down the workflow to an Image Viewer (**d**) and, as a subset of data points, to the MDS widget that performs multi-dimensional scaling (**e**). Just like in Fig. 1, the MDS widget shows a planar projection of data points (images) and highlights, in this case, the image points selected in the Confusion Matrix. Altogether, the components of this workflow are used to quantitatively evaluate the expected performance of machine-learning models through cross-validation and to support further exploration of correctly and incorrectly classified images

The meta information on the timing of bone healing, type of mouse oocyte, protein localization in yeast and developmental phase in *Dictyostelium* helped us interpret the results but was not used in model inference in the unsupervised data mining. It is also possible to use this information explicitly to build models that predict these phenotype classes using supervised data mining. Before using the models for prediction (see corresponding workflow in the Supplementary Note 6), we can assess their accuracy by learning from a training set and testing the models on a separate test set. The workflow in Fig. 3 performs these tasks and uses cross-validation for accuracy assessment. We used logistic regression for modeling from the image embedding matrix, and cross-validation for estimating the accuracy. Only five out of the 131 images from the oocyte phenotyping were misclassified, resulting in a surprisingly high accuracy of 96% (see Supplementary Table 1). To compare our approach to manual analysis, we presented the same 131 oocyte images to three reproductive biologists during their training period. These biologists had different levels of training experience (i.e., beginner, medium, and good) and their classification accuracy, compared to that of the expert, ranged from 78.7 to 84.5%^19^, quite lower than the accuracy of the automated approach.

In addition to providing high accuracy, the workflow shown in Fig. 3 helps the biologist to explore and gain understanding through the analysis of misclassifications in an interactive confusion matrix. For example, the workflow can show the correctly classified SN oocytes in MDS and in the image viewer. The same workflow was applied to the other three image sets. It resulted in high cross-validated accuracy of logistic regression for the phenotype classification of bone healing (F1 = 0.95), *Dictyostelium* development (F1 = 0.82), and yeast protein localization (F1 = 0.95) (see corresponding workflows in the Supplementary Table 1). We compared the performance of this workflow to image classification using features from more traditional image analytics pipelines. Features extracted by a pipeline in CellProfiler^20^ and scale-invariant feature transform^21^ were less informative and yielded cross-validation accuracies that were consistently below those obtained with image embedding by deep models (see Supplementary Table 3).

### Access to different image embedders

Embedding is the process of passing the image through an existing deep network to acquire its vector representation. The Image Embedder widget in Orange can accommodate different embedders (see Supplementary Note 5 for a current list) and it can be extended with specialized embedders when those become available. For example, in Google’s InceptionV3^6^—a convolutional 48-layered neural network that was trained on 1.2 million images from the ImageNet repository—the penultimate layer consists of 2048 nodes, and thus every input image is embedded into a 2048-element vector. The actual embedding process is either carried out on a dedicated server (the default option that uses InceptionV3) or runs locally. For local embedding, we use SqueezeNet, a deep-convolutional network that along with accuracy also optimized network complexity^22^. The advantage of SqueezeNet is that the images stay local on the user’s computer and are not sent to the server, thus also accommodating for privacy. We have not observed major differences between SqueezeNet’s embedding in accuracy when compared to other more complex networks like InceptionV3 across our four case studies (see Supplementary Table 1 for accuracy study). The use of server-based embedders may benefit from the speed of embedding in case of larger image sets, as well as provide a way to compare SqueezeNet to other, bigger and better-known trained deep models.

While we expect that specialized embedders will become available in the near future (e.g., for molecular and cell biology and pathology), our pilot study also suggests that general embedders might perform adequately well. For example, we found that cross-validated F1 accuracies were high when comparing expert annotations to image class predictions by logistic regression trained on features inferred from four different neural networks. The accuracy was around 0.95 for all cases except for the *Dictyostelium* phenotyping, which resulted in an F1 score around 0.82. Surprisingly accurate models were inferred from features by a deep network that was trained on 79,433 images of paintings^23^. These findings suggest that transfer learning, even in its most straightforward form, can be applied to a broad range of image analytics problems. They also suggest that the utility of domain-specific networks may be limited such that their marginal increase in accuracy may not justify the effort associated with generating them.

## Discussion

We present a tool and a free, open-source working environment for image analytics. The solution builds upon the visual programming data mining framework Orange, using the framework’s ability to construct workflows, develop data models, and engage in interactive visualizations. Modern image analytics are well supported in programming environments, such as those built around Python and enhanced with libraries for deep learning such as TensorFlow, PyTorch, and Keras. While these toolboxes should be preferred by any advanced user or data scientists, Orange aims to complement them by providing an accessible and interactive environment that still offers a high degree of functionality and can adapt to specific needs through visual programming and construction of problem-specific workflows.

Orange’s image analytics is intended for analysis of smaller image sets where the starting point is image embedding using a pre-trained deep network. Currently, we support a choice of frequently used deep network models, which provide good accuracy in the four cases we have studied in the paper, even though the embedders we use were not trained on images from molecular biology (see Supplementary Information). Orange embeddings rely on the penultimate layer of those deep networks, where transfer learning is achieved through encoding of images with features from this layer and is followed by using classical machine-learning method, such as random forests or logistic regression. Transfer learning could be achieved by partial or complete retraining of an existing network, but this task would require suitable hardware and may have an impact on the computational time. In designing a framework that avoids retraining the embedding part of the deep neural network, we aimed at a solution that is fast and can execute on a common computer or laptop. With this choice, we have potentially sacrificed accuracy that could arise from more complex solutions, but those solutions would be computationally more challenging and would require specialized hardware.

The framework we propose deals with images as a whole and relies on their embedding to support a wealth of machine-learning tasks like clustering, classification, regression, outlier detection, and dimensionality reduction. Our current implementation is somewhat limited, as it does not support other important image analytics tasks, notably image segmentation. These tasks would fit nicely within the framework of visual programming and interactive analytics and are good candidates for inclusion in further releases of our software.

The Orange framework with its extension for image analytics provides a user-friendly interface for unsupervised and supervised mining of images from various domains of biomedicine. It runs on standard computers and laptops and does not require specialized hardware. Through visual programming and construction of intuitive workflows, Orange supports domain experts (e.g., biologists) in a field where knowledge of computer science and programming used to be essential. With easy access to machine learning and its combination with interactive visualizations Orange aims to democratize data science.

## Methods

### Visual analytics

The proposed approach to image mining uses visual analytics^24^ that combines interactive visualizations and automated data analysis, including machine learning. Orange addresses all essential phases of visual analysis frameworks^25^ that include data loading and transformation, data visualization with user interaction, inference of data models, and model visualization. Orange implements components for visualization and data processing, and through visual programming supports data analysts to combine and link data analysis components and construct data analysis workflows. A typical workflow component receives the data, processes it, visualizes the result of processing, and outputs the results of the analysis or any selection of the user for further processing by a downstream component in the workflow. For instance, the widget for hierarchical clustering in Fig. 1 receives the pairwise distances between data items, constructs the corresponding clustering, visualizes it in a dendrogram, and outputs the data items that are associated with user-selected branches of the dendrogram. The outputs of Orange components are instantaneously modified upon any change in the input or by any selection made by the user, thus resulting in a visual analytics system where any change in the component triggers changes in the downstream components that subsequently update their results and corresponding visualizations of data and models.

### Transfer learning and embedding

For embedding, images are represented with feature vectors inferred by pre-trained deep-convolutional networks^6^. Orange provides an interface to several deep models for image classification from the Keras Python library (https://keras.io), including InceptionV3, VGG16, and VGG19 (see Supplementary Note 5 for a complete list of deep models used) and represents images from features of the penultimate layer of these networks. In InceptionV3, for example, an image is represented with 2048 features that are further processed by supervised or unsupervised machine learning in the workflows in Figs. 1 and 3.

### Machine learning

The workflows in Figs. 1 and 3 employ several standard data mining procedures such as computation of pairwise distances between data items, hierarchical clustering, and multi-dimensional scaling for model construction and cross-validation, and model scoring for model evaluation. Wherever possible, Orange embeds standard Python libraries for machine learning and data manipulation, including numpy (http://www.numpy.org), scipy (https://www.scipy.org) and scikit-learn^26^, and wraps their functionality within building blocks of workflows that provide an interface where the user can change the parameters of machine-learning methods or browse through results and related visualizations of the inferred models. In our workflows, we have used cosine distances between feature vectors, multi-dimensional scaling and hierarchal clustering with Ward linkage, and logistic regression. Default parameters of these methods were used unless otherwise noted.

### Reporting summary

Further information on research design is available in the Nature Research Reporting Summary linked to this article.

## Supplementary information

Supplementary Information

Reporting Summary

## Data Availability

Images of bone fraction repair, yeast protein localization, mouse oocytes, and development of social amoeba are available online at https://figshare.com/articles/Orange-Image-Analytics/9632276 (see Supplementary Note 1). These data can also be accessed through the Orange Datasets widget.

## Code availability

We recommend using the newest version of Orange available through the Orange website (http://orange.biolab.si). Orange version 3.22.0 used in this study is available at https://orange.biolab.si/download/files. Orange’s source code is available on GitHub (https://github.com/biolab/orange3). Components for image analytics are available in Image Analytics add-on; to install, use Options > Add-ons… and select Image Analytics. The source code for Image Analytics add-on is available on GitHub (https://github.com/biolab/orange3-imageanalytics); version 0.3.1 of the add-on was used to render data in the manuscript. The Supplementary Notes 4 and 6 include pointers to workflows that can be used to reproduce all the figures and results in this study.
