# Supplementary material for: Democratized image analytics by visual programming through integration of deep models and small-scale machine learning
Source: Nat Commun. 2019 Oct 7;10:4551. doi: 10.1038/s41467-019-12397-x (PMC6779910; doi:10.1038/s41467-019-12397-x)
Supplement: Supplementary file 1 — Supplementary Information [file 41467_2019_12397_MOESM1_ESM.pdf]

Supplementary Information

Democratized Image Analytics by Visual Programming through Integration of Deep Models and Small-Scale Machine Learning

Godec et al.

This is supplemental information for the manuscript published by Nature Communications that introduces an easily accessible tool to combine deep model-based image representation and machine learning in a framework for the analysis of smaller sets of biological and biomedical images. The tool relies on a visual programming approach to image analytics and uses Orange, a data mining suite and a set of components – widgets – for image visualization, vector representation, and mining. We show the applicability of the approach on analysis of images from bone healing, classification of developmentally competent or incompetent mouse oocytes, yeast protein localization and developmental phenotyping in a social amoeba. In this Supplement, we describe the image sets used in our case studies, provide instructions on how to load the image sets into Orange, define the data analysis workflows introduced in the letter, provide pointers on how to replicate the results of this analysis, and analyze classification accuracy using different deep learning models. All the data and the software we refer to are open and available for reuse.

Supplementary Note 1:
The Data

We used four different image sets to showcase the proposed transfer learning approach and illustrate the power of the proposed tool. Below, we provide background information on each data set. We also offer a link to a zipped file containing the images that can be loaded with Orange's Import Images widget, and a link to an Excel file that contains the list of images and annotations and that can be loaded into Orange using its File widget. See Supplementary Note 4 on loading the data for different means of importing the images and image annotations into Orange. In all case studies, the images were labeled with one of the classes, for which we provide the frequency statistics that report on how many images belonged to a specific class.

Bone Healing

Images: https://ndownloader.figshare.com/files/17282186 (also at http://file.biolab.si/images/bone-healing.zip)
Data with annotations: https://ndownloader.figshare.com/files/17282198 (also at http://file.biolab.si/datasets/bone-healing.xlsx)
Classes and class frequency for the total of 37 images: Day7 (19), Day14 (18).

Fracture repair is a multicellular and dynamic process regulated by complex cytokines and growth factors. Skeletal stem cells (SSCs) are a natural source of new osteoblasts and are critical for bone remodeling and fracture repair[1,2]. However, in vivo injury response of such cells in different ages or disease conditions has not been clearly defined. Due to cellular complexity and heterogeneity in injury sites, the detection of rare stem cells and how stem cells behave in early repair process has been technically challenging. We have previously developed in vivo imaging techniques to investigate the role of endogenous skeletal stem/progenitor cells

(SSPCs) and their progeny in bone repair. We then crossed Mx1/Tomato (a SSPC marker) mice with αSMA-GFP or Nestin-GFP mice (known mesenchymal cell markers) and tested if we could track Mx1-positive (Tomato) SSCs and αSMA-positive mesenchymal (GFP) cells in the injury in vivo. Using these stem cell-lineage tracing models and intravital imaging of induced microfractures in calvarial bone, SSCs at the injury sites were sequentially imaged seven days and 14 days after injury, in which critical events in the early repair process occur. In the reported work, we have analyzed 37 images (see Supplementary Figure 1) with bone healing phenotypes, split into two groups: 19 images at the 7th day of healing (D7) and 18 at the 14th day of healing (D14).

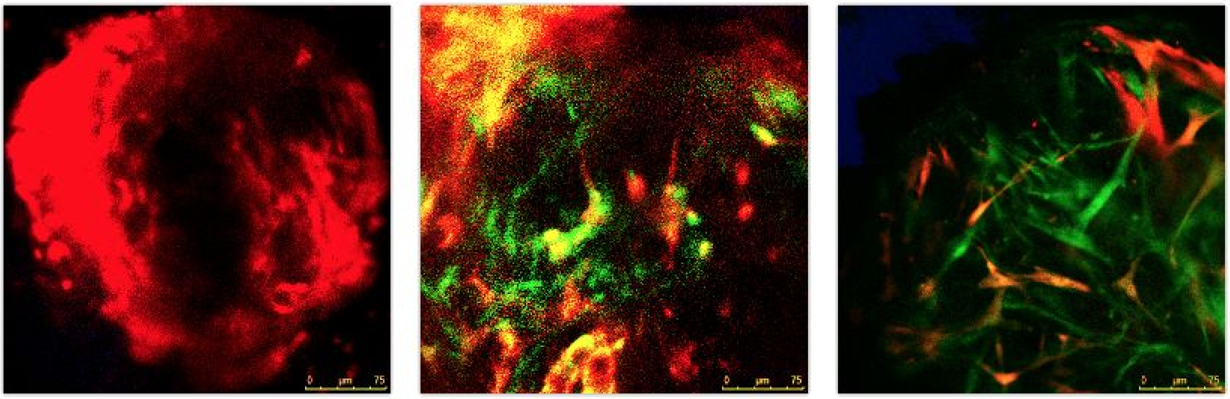

D14-Left injury 1_z110-m1 | D14-Left injury 4-2_z044-m1 | D7-I 4-3_z026

Supplementary Figure 1: Images from a bone-fracture repair that involves skeletal stem cells (SSCs). The images in this example are from mice that were the progeny of a cross between mice carrying Mx1/Tomato (red), which is a skeletal stem/progenitor cells (SSPCs) marker, and mice carrying αSMA-GFP or Nestin-GFP (green), which are mesenchymal cell markers. The bones were injured, and images were taken in vivo seven days (image on the right) and 14 days after injury (image on the left and central image), when critical events in the early repair process occur. The labels below the images correspond to the file names in the supplemental data.

Mammalian Oocyte Developmental Competence

Images: https://ndownloader.figshare.com/files/17282189 (also at http://file.biolab.si/images/oocyte-competence.zip)
Data with annotations: https://ndownloader.figshare.com/files/17282201 (also at http://file.biolab.si/datasets/oocyte-competence.xlsx)
Classes and class frequency for the total of 131 images: NSN (62), SN (69).

Female infertility is a pathology that affects a growing number of women worldwide. According to the World Health Organisation, 15-20% of couples are infertile (40% being the female infertility contribution). Also, a third of women undergoing oncological treatment is at risk of premature ovarian failure (in numbers: 58,000/year or 280,000/year patients in Countries like Italy or USA, respectively). The identification of markers of the oocyte developmental

competence could contribute to improving Artificial Reproductive Technologies and, in turn, increase the overall pregnancy success rate of these women.

When stained with the supravital fluorochrome Hoechst 33342, mouse fully-grown antral oocytes display two major types of chromatin organization. A type shows a ring of Hoechst-positive heterochromatin surrounding the nucleolus, and for this reason, the oocyte is named Surrounded Nucleolus (SN) oocyte (Supplementary Figure 2A). The other type, referred to as not surrounded nucleolus (NSN) oocyte (Supplementary Figure 2B), has more dispersed chromatin lacking aheterochromatic ring. These two oocyte types are present in all mammals, including our species.

Several are the cytological and molecular differences between SN and NSN oocytes[3,4], but the most striking regards their different developmental competence. After isolation from the ovary and maturation *in vitro*, both reach metaphase II and following fertilisation develop to the 2-cell stage, but, while NSN oocytes always arrest at this stage, SN oocytes may complete development with the birth of a new individual[5].

In this study, we analysed a total of 131 fluorescence images of the nuclei of mouse fully-grown antral oocytes, 62 were SN and 69 NSN oocytes, as classified by three independent researchers.

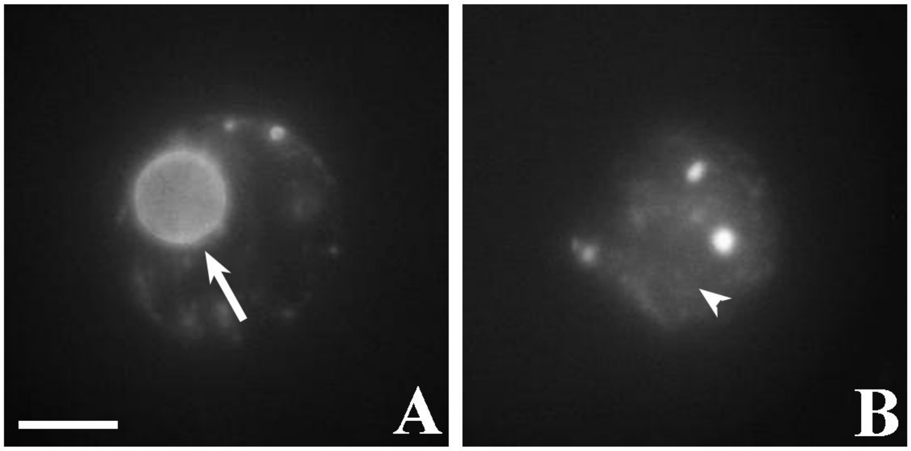

Supplementary Figure 2: Representative images of the nucleus of mouse fully-grown antral oocytes. A) Surrounded Nucleolus (SN) oocyte; image id SN_3. B) Not Surrounded Nucleolus (NSN) oocyte; image id NSN_42. Arrow, ring of Hoechst-positive heterochromatin surrounding the nucleolus; Arrowhead, a nucleolus lacking the heterochromatic ring. Bar, 10 μm.

Yeast Protein Localization

Images: https://ndownloader.figshare.com/files/17282192 (also at http://file.biolab.si/images/yplp.zip)
Data with annotations: https://ndownloader.figshare.com/files/17282204 (also at http://file.biolab.si/datasets/yplp.xlsx)

Classes and class frequency for the total of 2,569 images: cytoplasm (1,244), nucleus (517), mitochondria (437), endoplasmic reticulum (ER) (218), cellular periphery (62), punctate (54), spindle (37).

We have considered yeast protein localization images from YPL+.db (http://yplp.uni-graz.at), an annotated localization image database maintained at the University of Graz. This database contains information on the subcellular localization of the majority of proteins in *Saccharomyces cerevisiae* yeast cells, obtained by in vivo high-resolution confocal imaging (Oskolkova, Leitner and Kohlwein, personal communication). The YPL+.db database includes images of localization of 3,533 proteins and reports on up to three different location sites for each of the proteins (see Supplementary Figure 3). For our case study, we considered only 2,622 proteins with single-site localization, and from these included only localization sites with more than 30 reported proteins. The resulting dataset contains 2,569 proteins and their localization images.

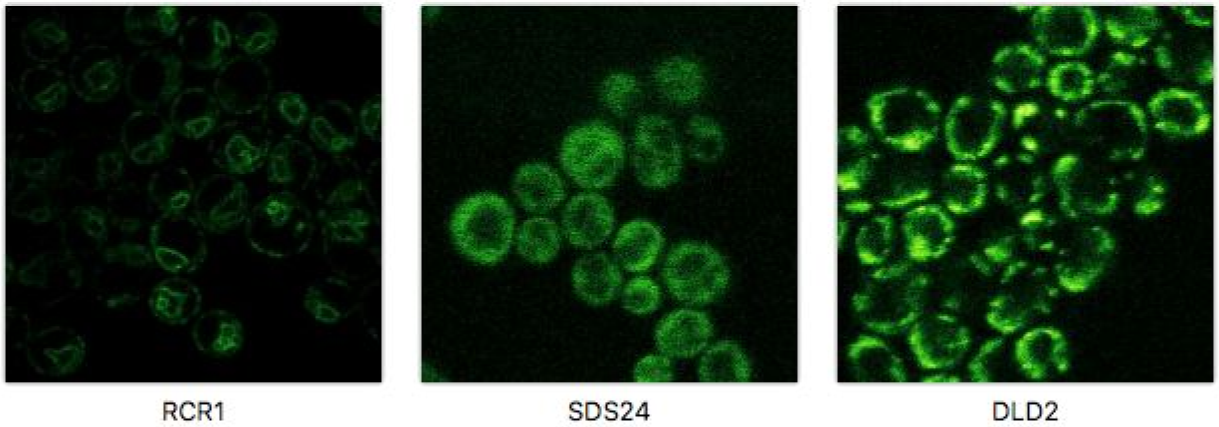

Supplementary Figure 3. Images with localization of three different proteins from the YPL+ database. Protein Rcr1 (encoded by *RCR1*) is localized in the endoplasmic reticulum, Sds24 (encoded by the gene *SDS24*) is localized in the cytosol, and the protein Dld2 (encoded by *DLD2*) is localized in the mitochondria.

Development of Social Amoebae

Images: https://ndownloader.figshare.com/files/17282195 (also at http://file.biolab.si/images/dicty-development.zip)
Data with annotations: https://ndownloader.figshare.com/files/17282207 (also at http://file.biolab.si/datasets/dicty-development.xlsx)
Classes and class frequency for the total of 152 images: STR (20), LAG (68), TAG (64).

We have considered images of strains of the social amoeba *Dictyostelium discoideum*[6] (see Supplementary Figure 4). The strains were generated by chemical mutagenesis in a study aimed at developing a method for identifying mutated genes by whole-genome sequencing. The cells were grown in submerged culture and induced to develop on buffered-agar plates by starvation. Under these conditions, the cells aggregate into multicellular structures. We captured

the images by photography through a dissecting microscope at different times during the 24-hour developmental program. From 233 images of strains at different developmental stages we considered three consecutive developmental morphologies: streams (20 images), loose aggregates (68 images) and tipped aggregates (64 images). Since development is not entirely synchronous, the images may contain mixtures of structures at different stages. The biologists usually determine the stage by the majority phenotype, but this classification is somewhat subjective. Moreover, some mutant strains may exhibit morphologies that are not typically found in the wild type (neomorphs). An objective means of classification would be desirable.

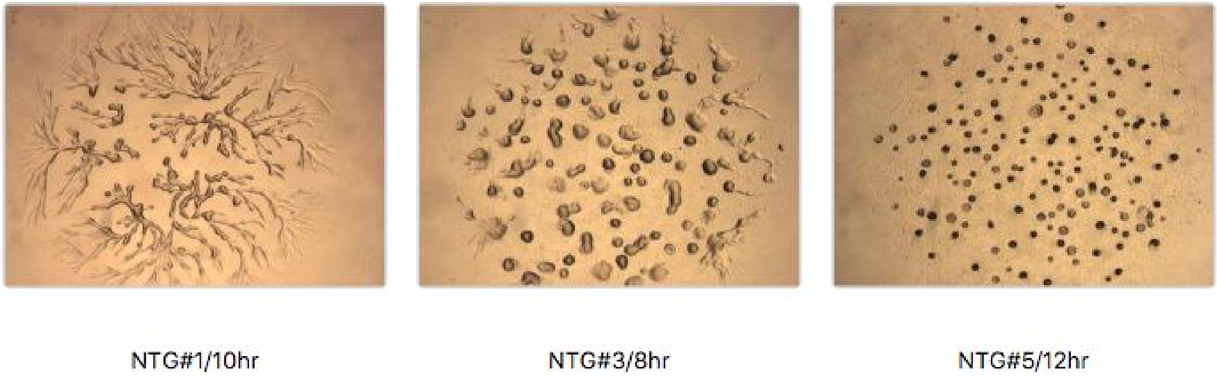

Supplementary Figure 4: Images of development of a colony of a social amoebae. Development of three different strains at three different times are shown, where a colony of strain NTG#1 is at the developmental stage of streams, NTG#3 at the stage of loose aggregates, and NTG#5 is at the stage of tipped aggregates. The labels below the images correspond to the file names in the supplemental data.

Supplementary Note 2:
Installation of Orange Data Mining Software

1. Install Orange Data Mining software from http://orange.biolab.si. Installation packages are available for Windows, Mac OSX and Linux operating systems.
2. Run Orange (watch the Welcome to Orange YouTube video for introduction to basic functions). Then install the Image Analytics Add-on by selecting Options->Add-ons… from the main menu, checking the checkbox in front of Image Analytics and pressing ok (Supplementary Figure 5).

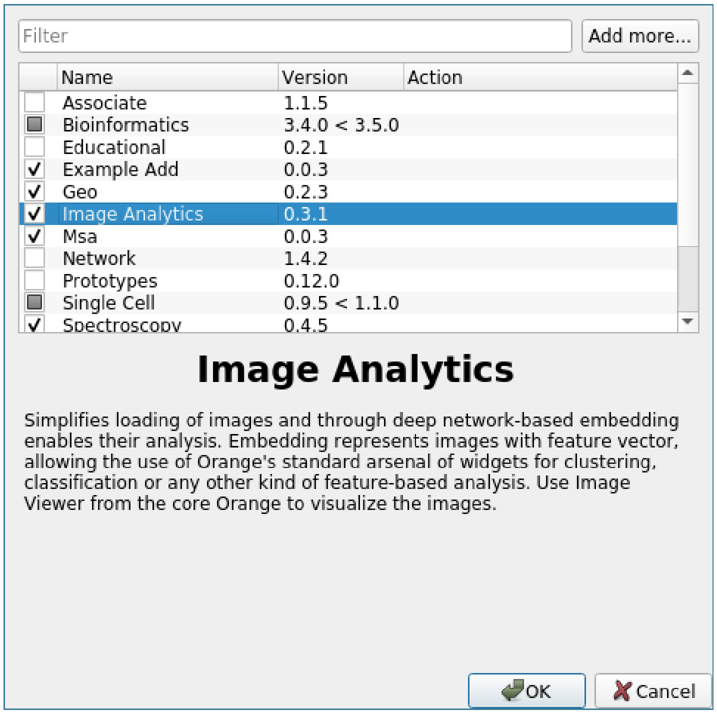

Supplementary Figure 5: Installation of Image Analytics add-on to Orange Data Mining software requires opening of a window with a list of add-ons from the Options menu (Options -> Add-ons…).

Optionally check any of the following videos to familiarize yourself with visual programming and image analytics in Orange:

- Getting Started with Orange 14: Image Analytics - Clustering
- Getting Started with Orange 15: Image Analytics - Classification

Supplementary Note 3: Hardware Requirements

None. Orange runs on most standard personal or laptop computers with OS X, Windows or Linux operating systems. The tool uses already trained deep convolutional neural networks and hence only runs them in feed-forward way to obtain image embeddings. Embedding takes places on the server for all but SqueezeNet networks, which is optimized for speed and can be executed on a CPU of a local machine. No special hardware is needed to run Orange image analytics.

Supplementary Note 4:
Loading the Image Data

We describe three ways of importing images from the four case studies included in our manuscript. The first involves the Data Sets widget, which connects to a repository of datasets that include the four data tables with image information from our manuscript, and includes links to the images on our image server. Images can also be loaded into Orange by importing them from a local directory that contains image files, or by loading a data table that has references to local or remote image files. We have prepared the data from our case studies in all of these formats.

Loading Case Studies from the Data Sets Widget

Use the Data Sets widget and select any of our four case studies. The studies have been tagged with the keyword “image analytics” and are short-listed, together with other examples image sets, upon entering this keyword in the filter of the Data Sets widget.

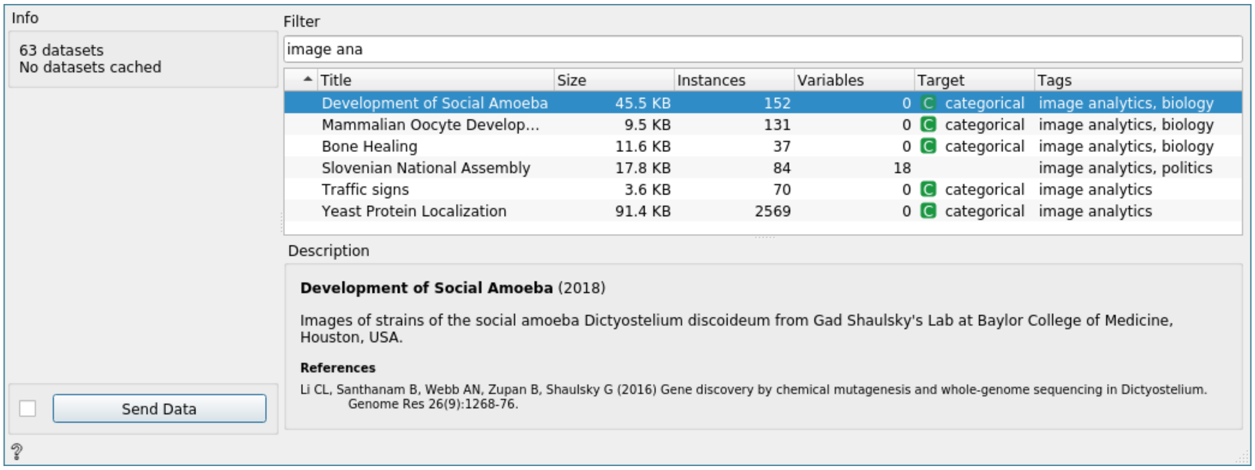

Supplementary Figure 6: The Datasets widget provides a list of ready-to-use datasets and includes the four data sets with images from our case studies. Use the keyword “image analytics” to retrieve them, select the dataset and press Send Data to push the data to the output of the Data Sets widget.

The Data Sets widget loads a data table that includes a description of the images and links to the images on the image server. These data can be inspected in the Data Table widget. Images contained in the data can be shown in the Image Viewer. Below is an example of a workflow containing these widgets. If this is your first time to use Orange, you can learn about widgets and visual programming in the first few short videos on Orange’s YouTube channel.

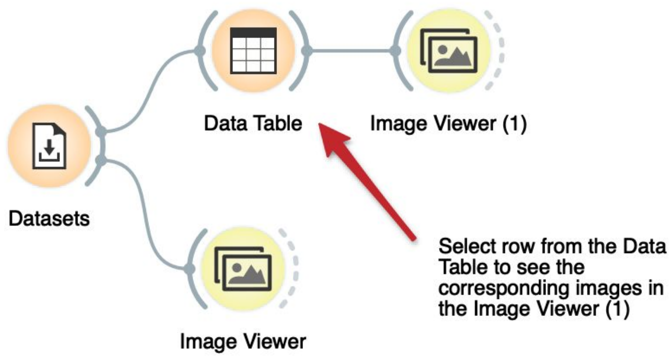

Supplementary Figure 7: Workflow with the Data Sets widget. The workflow definition file is available here. Download it and load it into Orange with a File->Open command. Double click on any of the widget to see their content, or double click on Image Viewer to inspect the list of included images.

Importing Images from Image Folders

1. Download any of the zipped files that contain a folder with images (bone-healing.zip, oocyte-competence.zip, yplp.zip, dicty-development.zip). Uncompress the file. The compressed file contains a directory with subdirectories that correspond to image classes and contain raw images.
2. Use the Import Images widget to locate and open a directory that holds the images. You can check if the data were loaded appropriately by displaying the list of images in the Data Table widget, or checking out the images in Image Viewer.

An Orange screenshot with the workflow that loads the images from the local data folder and then uses Image Viewer to inspect the images and Data Table to check the format of the loaded data is shown in Supplementary Figure 8.

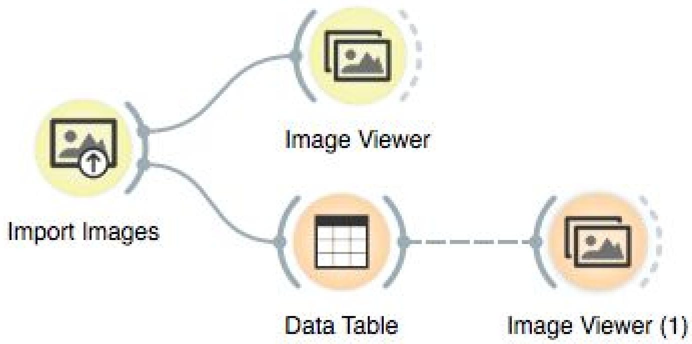

Supplementary Figure 8: A workflow that enables importing of images from the local directory.

Notice that Image Viewer will display all the images from the input data folder, while Image Viewer (1) displays only the images that correspond to selected data rows in the Data Table. In

the workflow above, we have not selected any rows in Data Table yet, hence the line that connects the output of the Data Table with the input of the Image Viewer is dashed.

Importing Images by Loading the Data with Image Descriptions

1. Download any of the Excel files that contain a list of images with descriptions (bone-healing.xlsx, oocyte-competence.xlsx, yplp.xlsx, dicty-development.xlsx).
2. Load these files using the File widget, and explore the data table or the images included in the workflow from Supplementary Figure 9.

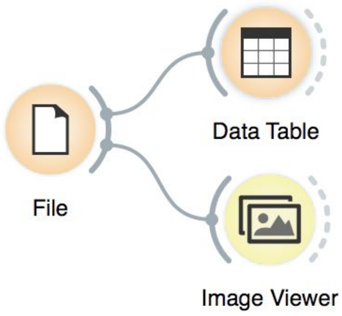

Supplementary Figure 9: Excel files with lists and descriptions of images can be loaded using the File widget (double click to open the widget, then select the input file).

Supplementary Note 5: Embedders

Orange image analytics currently includes six different embedders, of which five are implemented on the embedding server and one runs on a local computer:

- InceptionV3 is Google's deep neural network for image recognition which consists of 48 layers[7]. It is trained on the ImageNet data set and performs with 5.6% top-5 error at the ImageNet dataset. We use pre-trained networks implementation from the Tensorflow's models repository (https://github.com/tensorflow/models).

- VGG16 is a deep neural network for image recognition proposed by Visual Geometry Group from the University of Oxford which consists of 23 layers[8]. It is trained on the ImageNet data set and performs with 7.4% top-5 error at the ImageNet dataset. We use a community implementation of the network in Tensorflow with original weights (https://github.com/machrisaa/tensorflow-vgg).

- VGG19 is a deep neural network for image recognition proposed by Visual Geometry Group from the University of Oxford which consists of 26 layers[8]. It is trained on the ImageNet data set and performs with 7.3% top-5 error at the ImageNet dataset. We use

a community implementation of the network in Tensorflow with original weights (https://github.com/machrisaa/tensorflow-vgg).

- SqueezeNet is a deep model for image recognition that achieves AlexNet-level accuracy on ImageNet with 50x fewer parameters[9]. The network consists of 26 layers. We re-implemented the SqueezeNet by using weights from the author's pretrained model.
- Painters is a model trained to predict patients from artwork images[10]. The network consists of 24 layers and was trained on the dataset from Painter by Numbers competition on Kaggle (https://www.kaggle.com/c/painter-by-numbers). We use the pretrained author's implementation.
- DeepLoc is a convolutional network trained on 21,882 images of single cells that were manually assigned to one of 15 localization compartments[11]. The network consists of 11 layers. In our implementation, we use the pre-trained network proposed by authors.

Inception v3[7] is just one of the convolutional neural networks available for feature-based representation of images offered by the Image Embedding widget. At the time of submission of this work, the Image Embedding widget included other embedders, including VGG16, and VGG19[8], and SqueezeNet[9]. Both VGG16 and VGG19 were all pre-trained on a collection of images from ImageNet. Image Embedding also includes Painters, an embedder that was trained on 79,433 images of paintings by 1,584 painters and won Kaggle's Painter by Numbers competition[10]. SqueezeNet computes embeddings locally on users computer since the network is small while other embedders compute the vector representation at the server.

Embedding server uses Kubernetes cluster with eight dedicated computational nodes. Each embedder runs in a Docker container. The number of containers are scaled according to the load. Currently, Google's Inception v3 is the most popular embedder and uses 90% of computational resources. Image Embedding widget locally scales the images to fit the input size of each deep network, sends the scaled images in batches to image embedding cluster, and receives the embedding.

Supplementary Note 6: Example Workflows

Example Workflow for Unsupervised Data Mining

The workflow for unsupervised image mining from Figure 1 of the main manuscript is displayed in Supplementary Figure 10. The workflow definition file is available here. To load it into Orange, download the file and use File->Open to select the workflow file. The workflow loads images on bone healing. To change the images set, select any of the image data sets available in the Data Sets widget (see section Loading Case Studies from Data Sets Widget of this document for

details). Notice that Data Sets also includes example data files that do not include images and on which the workflow on image analytics cannot be used.

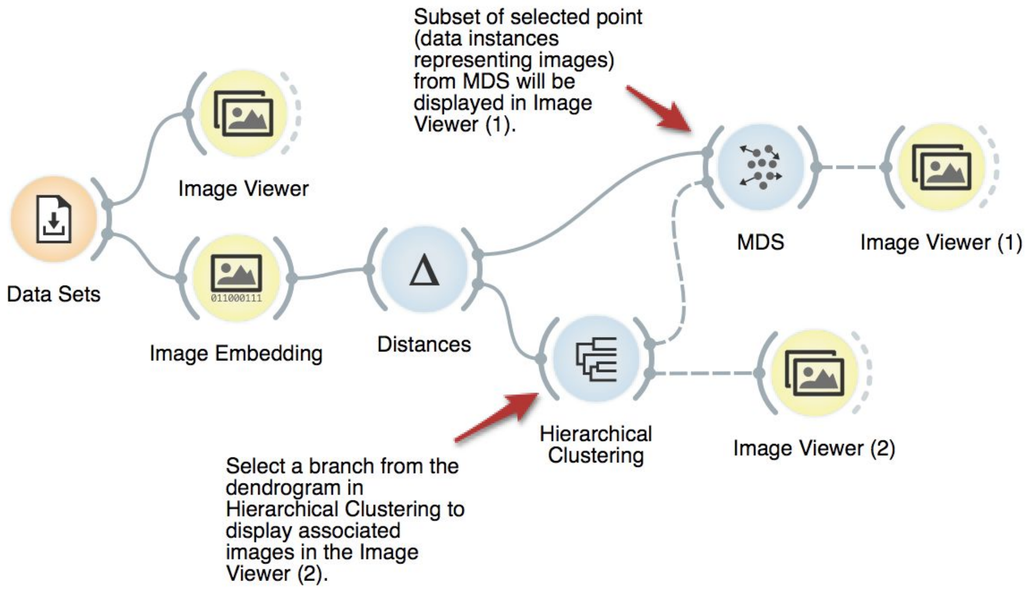

Supplementary Figure 10: An unsupervised image mining workflow from Figure 2 of the main manuscript. Images are first embedded into vector spaces, compared to each other by computing cosine similarity between image vector pairs, and then fed into hierarchical clustering and multi-dimensional scaling. Images from any selection of a branch in the hierarchical clustering dendrogram are displayed in Image Viewer (2) and fed to MDS, where they are shown as a subset of the data points displayed in this two-dimensional visualisation. Images for selection of data points from MDS are visualized in Image Viewer (1).

Example Workflow for Supervised Data Mining

A workflow for image classification from Figure 3 of the main manuscript is displayed in Supplementary Figure 11. The workflow definition file is available here. To load it into Orange, download the file and use File->Open to select the workflow file. The workflow loads images from mammalian oocytes that belong to one of the two classes. To change the image set, select any of the image data sets available in the Data Sets widget (see section Loading Case Studies from Data Sets Widget of this document for details).

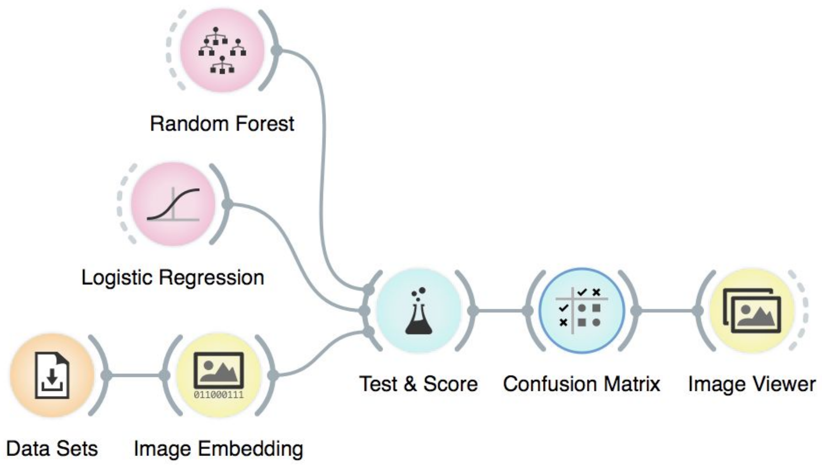

Supplementary Figure 11: A workflow that estimates the quality of classification of images. This is the supervised part of the workflow from Figure 3 of the main manuscript. Images are first embedded into vector spaces. Besides logistic regression, this workflow compares and evaluates the performance of a random forest using 10-fold cross-validation. Correctly or incorrectly classified images can be selected in the Confusion Matrix widget and displayed in the Image Viewer widget.

Supplementary Note 7: Quantitative Comparisons

Comparison of Deep Learning-Based Vector Representation of Images

We have compared the vector-based presentation of the five standarder embedders currently included in Orange image analytics through cross-validated accuracy of logistic regression, which aimed to predict the class of a given image. We would assume that better vector presentation of images would yield higher accuracy scores. The results of this experiment, using a selected accuracy measure (F1) that had the largest spread of values across different settings is provided in Supplementary Table 1.

The results show good performance of all four embedders, and also show that classification of the social amoeba phenotypes is the hardest of the four classification tasks. We were surprised at the good performance of Painters, the network trained on a relatively smaller collection of paintings.

Supplementary Table 1: Ten-fold cross-validated harmonic averages of the precision and recall (F1) scores of L2-regularized logistic regression (regularization parameter C was set to 1) on the four image sets (see The Data section) and using various pre-trained deep neural networks for vector representation. Note that the results may slightly vary and depend on the order of the images in the input data set and hence the composition of the data subsets in cross-validation.

	Embedder				
Data set (number of images/classes)	Inception v3	VGG16	VGG19	Painters	Squeeze Net
Bone healing (37/2)	0.946	1.000	0.892	0.891	0.919
Mammalian oocyte development competence (131/2)	0.962	0.939	0.977	0.985	0.985
Yeast Protein Localization (2569/7)	0.952	0.934	0.926	0.957	0.915
Development of Social Amoeba (152/3)	0.820	0.828	0.781	0.855	0.856

Comparison of General-Purpose Deep Models to Yeast Protein Localization-Trained DeepLoc

We compared the value of image vectorization of Inception v3 and DeepLoc[11]. DeepLoc is a convolutional network trained on 21,882 images of single cells that were manually assigned to one of 15 localization compartments. The authors also provide a testing set of 4,516 images (see Supplementary Figure 12). The images from additional two classes “ghost” and “dead” were removed, resulting in 4,241 images in the dataset (http://file.biolab.si/images/deeploc-test.zip). We used DeepLoc for embedding, again considering 512 features from the DeepLoc network penultimate layer. We compared this feature set with features obtained through image embedding by Inception v3 in a similar setting as in the previous section. In the comparison, we measured the cross-validated accuracy of the logistic regression (Supplementary Table 2).

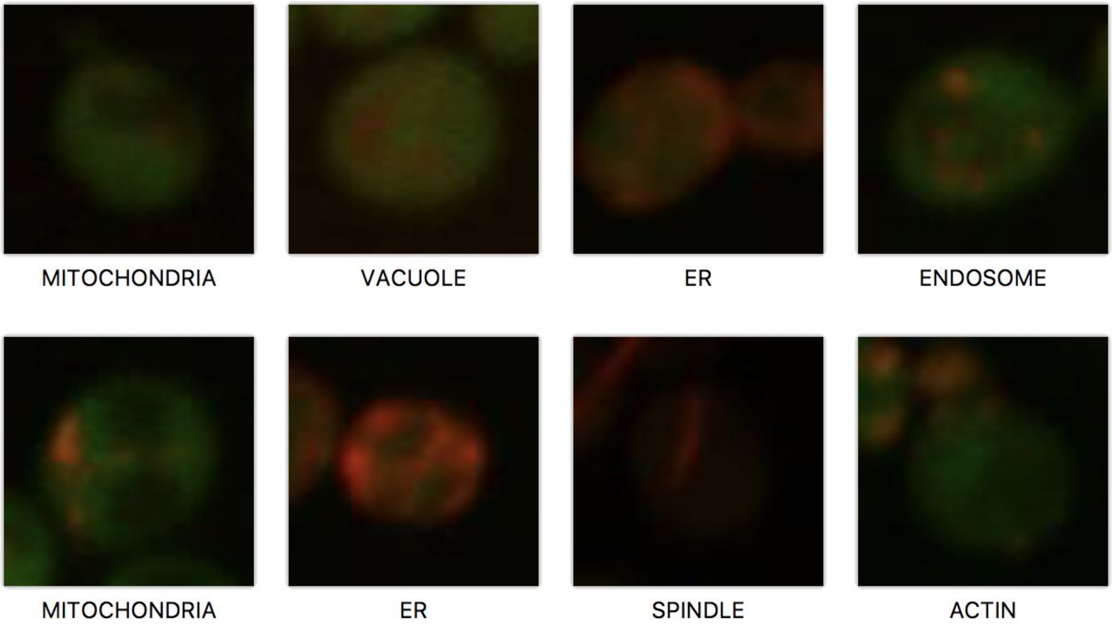

Supplementary Figure 12: A set of images from the test dataset from Kraus et al.[11] with the name of the associated localization compartment. The images in the training set for DeepLoc are of similar types.

Supplementary Table 2: Ten-fold cross-validated harmonic average of the precision and recall (F1) scores of L2-regularized logistic regression (regularization parameter C was set to 1) on two yeast protein localization image sets using image vectorization from a model that was trained on a specific set of yeast localization images (DeepLoc) and a general-purpose model (InceptionV3; results with the other three deep networks examined in the previous section were similar). Note that the results may slightly vary and depend on the order of the images in the input data set and hence the composition of the data subsets in cross-validation.

	Embedder	
Data set	DeepLoc	InceptionV3
DeepLoc Test Set (Kraus et al., 2017)	0.841	0.674
Yeast Protein Localization (YPL+.db, this supplement)	0.777	0.952

The images from Kraus et al.[11] exemplified in Supplementary Figure 12 were harder to classify than the yeast protein localization images from the YPL+.db repository we described above. The classification accuracy on the DeepLoc-specific images is, as expected, better with embedding by the DeepLoc model that used images of similar type in the training data set. Specialized models should, in general, provide better embedding than general-purpose models or models trained on different sets of images. Yet, their disadvantages is that a large image set is required for their training. In addition, they may be limited in scope, as illustrated when classifying yeast protein localization images from YPL+.db. In this case, DeepLoc embedding performed worse

than InceptionV3. DeepLoc performed similarly worse on the other three image sets from this supplement (data not shown).

Comparison to Image Profiles by CellProfiler and Bag of Words Model

We have compared image embedding by InceptionV3, which was among the best embedders from Supplementary Table 1, with images features that were inferred by CellProfiler[12] and the bag of visual words model.

CellProfiler was designed to quantitatively measure individual features from microscopy images and relies on image processing pipelines that are designed for a specific problem domain. For our set of images, we have constructed a CellProfiler pipeline (http://file.biolab.si/files/cell-profiler-feature-construction-pipeline.cppipe) that convert images to grayscale and infers 68 features that measure the granularity which is the spectra of size measurements of the textures in the image and texture that includes the degree and nature of textures within images. For each of our four image collections we have used this pipeline to prepare a data set (http://file.biolab.si/files/cell-profiler-image-data.zip) that includes image reference and class and CellProfiler features.

A bag of visual words model generates features for images with occurrence counts of a vocabulary of local image features. Local points in images and their features are retrieved using scale-invariant feature transform (SIFT)[13] and transformed into the bag of words vectors with a codebook generated using k-means clustering. In this work, the implementation from the OpenCV library[14] has been used. Since a codebook is generated on the training data, the generated features depend on the training set and are different for every iteration of cross validation. Instead of the generated data, we thus provide the Python script that we have used for the evaluation (http://github.com/PrimozGodec/imageanalytics-bag-of-words). We retrieve 128 dimensional vector for each key-point in the image with SIFT algorithm. The key-points over the training set are clustered in 50 clusters-visual words. Each image is therefore described with counts of key-point per cluster, and thus described with 50 features.

In Supplementary Table 3 cross-validated F1 scores of logistic regression on these data sets are compared to scores where InceptionV3 was used as an embedder. Notice that accuracy of models that were developed from features constructed by deep models resulted in consistently better accuracy. CellProfiler image processing pipelines run much longer then feed-forward feature construction by deep neural network: on average, it takes about 9 seconds to process an image with a pipeline used in Supplementary Table 3, compared to 2.3 seconds for InceptionV3, both running times assessed on single-core architecture. Notice that for the largest of our case studies (yeast protein localization) the post-processing of image profiles by clustering or training the classifier in Orange takes only a few seconds.

Supplementary Table 3: Ten-fold cross-validated harmonic averages of the precision and recall (F1) scores of L2-regularized logistic regression (regularization parameter C was set to 1) on the four image sets (see The Data section) where image features were constructed either by CellProfiler and InceptionV3 (a reference, results copied from Supplementary Table 1).

	Feature Constructor		
Data set (number of images/classes)	CellProfiler	Bag of words	InceptionV3
Bone healing (37/2)	0.837	0.334	0.946
Mammalian oocyte development competence (131/2)	0.954	0.861	0.962
Yeast Protein Localization (2569/7)	0.805	0.736	0.952
Development of Social Amoeba (152/3)	0.710	0.606	0.820

Supplementary Note 8: Authors and Their Contributions

Primož Godec* - software developer (Orange Image Analytics), server maintenance
Matjaž Pančur* - embedding server architect
Nejc Ilenič* - developer of early version of server-side embedders
Andrej Čopar - maintenance and administration of the server
Martin Stražar - testing, data preparation
Aleš Erjavec - software developer (Orange Image Analytics)
Ajda Pretnar - testing, data preparation, software documentation, video production
Janez Demšar - Orange software architect, developer
Anže Starič - Orange software project manager
Marko Toplak - Orange software developer, UI design
Lan Žagar - application testing, issue resolution manager
Jan Hartman - software developer (Orange Image Analytics)
Hamilton Wang - bone-fracture repair case study
Riccardo Bellazzi - antral oocytes in mice case study
Uroš Petrovič - yeast protein localisation case study
Silvia Garagna - antral oocytes in mice case study
Maurizio Zuccotti - antral oocytes in mice case study
Dongsu Park - bone-fracture repair case study
Gad Shaulsky - Dictyostelium development case study, manuscript writing
Blaž Zupan - conceived the software architecture, manuscript writing

* These authors contributed equally

Supplementary References

1. Méndez-Ferrer, S. *et al.* Mesenchymal and haematopoietic stem cells form a unique bone marrow niche. *Nature* **466**, 7308:829-34 (2010).
2. Park, D. *et al.* Endogenous bone marrow MSCs are dynamic, fate-restricted participants in bone maintenance and regeneration. *Cell Stem Cell* **10**, 3:259-72 (2012).
3. Tan, J.H. *et al.* Chromatin configurations in the germinal vesicle of mammalian oocytes. *Mol. Hum. Reprod.* **15,** 1:1-9 (2009).
4. Zuccotti, M. *et al.* What does it take to make a developmentally competent mammalian egg? *Hum. Reprod. Update* **17**, 4:525-40 (2011).
5. Inoue, A. *et al.* Contribution of the oocyte nucleus and cytoplasm to the determination of meiotic and developmental competence in mice. *Hum. Reprod.* **23**, 6:1377-84 (2008).
6. Li, C.L. *et al.* Gene discovery by chemical mutagenesis and whole-genome sequencing in Dictyostelium. *Genome Res.* **26**, 9:1268-76 (2016).
7. Szegedy, C. *et al.* Rethinking the inception architecture for computer vision. Proceedings of the IEEE Conference on Computer Vision and Pattern Recognition: 2818-2826 (2016).
8. Simonyan, K., Zisserman, A. Very Deep Convolutional Networks for Large-Scale Image Recognition, arXiv:1409.1556 (2014).
9. Iandola, F.N. SqueezeNet: AlexNet-level accuracy with 50x fewer parameters and <0.5MB model size. arxiv: 1602.07360 (2016).
10. Ilenič, N. Deep models of painting authorship, MSc Thesis, University of Ljubljana (2017).
11. Kraus, O.Z. Automated analysis of high-content microscopy data with deep learning. *Molecular Systems Biology*, **13**, 4:924 (2017).
12. Carpenter, A.E. CellProfiler: image analysis software for identifying and quantifying cell phenotypes. *Genome Biology* **7**:R100 (2006).
13. Lowe, D.G. Object recognition from local scale-invariant features. Proceedings of the International Conference on Computer Vision. **2**:1150-1157 (1999).
14. Bradaski, G., Kaehler, A. The OpenCV Library. *Dr. Dobb's Journal of Software Tools* 3 (2000).
