# Supplementary material for: Democratized image analytics by visual programming through integration of deep models and small-scale machine learning
Source: Nat Commun. 2019 Oct 7;10:4551. doi: 10.1038/s41467-019-12397-x (PMC6779910; doi:10.1038/s41467-019-12397-x)
Supplement: Supplementary file 2 — Reporting Summary [file 41467_2019_12397_MOESM2_ESM.pdf]

nature research

Corresponding author(s): Blaž Zupan

Last updated by author(s): Aug 14, 2019

Reporting Summary

Nature Research wishes to improve the reproducibility of the work that we publish. This form provides structure for consistency and transparency in reporting. For further information on Nature Research policies, see Authors & Referees and the Editorial Policy Checklist.

Statistics

For all statistical analyses, confirm that the following items are present in the figure legend, table legend, main text, or Methods section.

n/a	Confirmed	
☐	☒	The exact sample size (*n*) for each experimental group/condition, given as a discrete number and unit of measurement
☐	☒	A statement on whether measurements were taken from distinct samples or whether the same sample was measured repeatedly
☒	☐	The statistical test(s) used AND whether they are one- or two-sided<br>*Only common tests should be described solely by name; describe more complex techniques in the Methods section.*
☒	☐	A description of all covariates tested
☒	☐	A description of any assumptions or corrections, such as tests of normality and adjustment for multiple comparisons
☒	☐	A full description of the statistical parameters including central tendency (e.g. means) or other basic estimates (e.g. regression coefficient) AND variation (e.g. standard deviation) or associated estimates of uncertainty (e.g. confidence intervals)
☒	☐	For null hypothesis testing, the test statistic (e.g. *F*, *t*, *r*) with confidence intervals, effect sizes, degrees of freedom and *P* value noted<br>*Give P values as exact values whenever suitable.*
☒	☐	For Bayesian analysis, information on the choice of priors and Markov chain Monte Carlo settings
☒	☐	For hierarchical and complex designs, identification of the appropriate level for tests and full reporting of outcomes
☒	☐	Estimates of effect sizes (e.g. Cohen's *d*, Pearson's *r*), indicating how they were calculated

Our web collection on statistics for biologists contains articles on many of the points above.

Software and code

Policy information about availability of computer code

Data collection	Data used in the paper is comprised of plain image files. No specific software was used for data collection.
Data analysis	A version of Orange 3 (version: 3.13.0) used in this study is available at https://orange.biolab.si/download/files.

For manuscripts utilizing custom algorithms or software that are central to the research but not yet described in published literature, software must be made available to editors/reviewers. We strongly encourage code deposition in a community repository (e.g. GitHub). See the Nature Research guidelines for submitting code & software for further information.

Data

Policy information about availability of data

All manuscripts must include a data availability statement. This statement should provide the following information, where applicable:

- Accession codes, unique identifiers, or web links for publicly available datasets
- A list of figures that have associated raw data
- A description of any restrictions on data availability

Images of bone fraction repair, yeast protein localization, mouse oocytes, and development of social amoeba are available online at https://figshare.com/articles/Image_Analytics_by_Visual_Programming_through_Integration_of_Deep_Models_and_Small-Scale_Machine_Learning/9612269.

Field-specific reporting

Please select the one below that is the best fit for your research. If you are not sure, read the appropriate sections before making your selection.

☒ Life sciences ☐ Behavioural & social sciences ☐ Ecological, evolutionary & environmental sciences

Life sciences study design

All studies must disclose on these points even when the disclosure is negative.

Sample size	The manuscript presents the approach to image analytics. Four image sets are provided to explain the utility of the approach: bone healing (37 images), oocyte development (131 images), yeast protein localization (2,569 images) and development of social amoeba (153 images).
Data exclusions	Images considered in case studies were subjects of related studies. No images from these studies were excluded.
Replication	We provide the data, access to open source software, and access to workflows that were used in the analysis and case studies.
Randomization	The results of machine learning procedures were evaluated by mens of cross-validation.
Blinding	Blinding was not relevant and does not apply to our study study. We describe approaches to exploratory data analysis and environment for machine learning.

Reporting for specific materials, systems and methods

We require information from authors about some types of materials, experimental systems and methods used in many studies. Here, indicate whether each material, system or method listed is relevant to your study. If you are not sure if a list item applies to your research, read the appropriate section before selecting a response.

Materials & experimental systems

n/a	Involved in the study
☒	☐ Antibodies
☒	☐ Eukaryotic cell lines
☒	☐ Palaeontology
☒	☐ Animals and other organisms
☒	☐ Human research participants
☒	☐ Clinical data

Methods

n/a	Involved in the study
☒	☐ ChIP-seq
☒	☐ Flow cytometry
☒	☐ MRI-based neuroimaging
